# Multidisciplinary treatment of retroperitoneal ectopic pregnancy: a case report and literature review

**DOI:** 10.1186/s12884-022-04799-5

**Published:** 2022-06-07

**Authors:** Hainan Xu, Dali Cheng, Qing Yang, Dandan Wang

**Affiliations:** Department of Obstetrics and Gynecology, Shengjing Hospital of China Medical University, No.36 Sanhao Street, Heping District, Shenyang, 110004 PR China

**Keywords:** Retroperitoneal ectopic pregnancy, Laparotomy, Case report, Review

## Abstract

**Background:**

Retroperitoneal ectopic pregnancy (REP) is an extremely rare type of ectopic pregnancy, with a total of less than 32 cases reported in the English literature. Early diagnosis of REP is very difficult and all treatments entail a high risk of life-threatening complications.

**Case presentation:**

A 29-year-old nulliparous woman presented a history of 50-day amenorrhea and 7-day upper abdominal pain without vaginal spotting. The serum beta-human chorionic gonadotropin (β-hCG) value was 65,004 m-international units per milliliter (mIU/mL), but no intrauterine gestational sac was found via transvaginal sonography (TVS). Then transabdominal ultrasonography (TAS) and abdominal contrast-enhanced computer tomography (CT) identified a retroperitoneal ectopic pregnancy (REP) tightly adjacent to the inferior vena cava and the abdominal aorta. After consultation from a multidisciplinary team, systemic methotrexate (MTX, intramuscular 20 mg daily for 5 consecutive days) combined with ultrasound-guided local potassium chloride solution injection into the gestational sac was scheduled firstly for the patient. However, serum β-hCG continued to increase and the patient experienced worsening abdominal pain. Laparotomy was performed jointly by a gynecologist and a vascular surgeon. During the operation, the gestational sac with fetal bud measuring about 4.5 × 4.0x3.0 cm, tightly adherent to the surface of inferior vena cava and the left side of abdominal aorta, was carefully dissociated out from the surrounding tissues and removed *en bloc*. Histopathology examination confirmed the diagnosis of REP. The patient recovered uneventfully and her serum β-hCG returned to normal range on the 23^th^ postoperative day.

**Conclusions:**

Considering the possibility of REP and combined radiological examinations, such as ultrasonography and CT, are crucial for the early diagnosis of this rare condition. A multidisciplinary team is necessary to treat REP.

## Background

Ectopic pregnancy is a major cause of maternal mortality and morbidity encountered in the first trimester [1]. Nearly all ectopic pregnancies (95%) are implanted in the fallopian tube, whereas only merely 1% of ectopic pregnancies are implanted in the abdominal cavity [2]. Retroperitoneal ectopic pregnancy (REP), in which the gestational sac is implanted in the retroperitoneal cavity of the pelvis and abdomen, refers to an extremely rare type of abdominal ectopic pregnancy [3]. Once a retroperitoneal gestational sac ruptures, it can cause a catastrophic hemorrhage, especially for those located close to large blood vessels [3–5]. Here we report a case of REP implanted on the surface of the inferior vena cava, as well as the abdominal aorta, which was successfully treated in a multidisciplinary team. In order to provide reference for clinical practice in the diagnosis and treatment of REP, we also conducted a review on all of the reported cases in English literature.

## Case presentation

**Fig.1:**
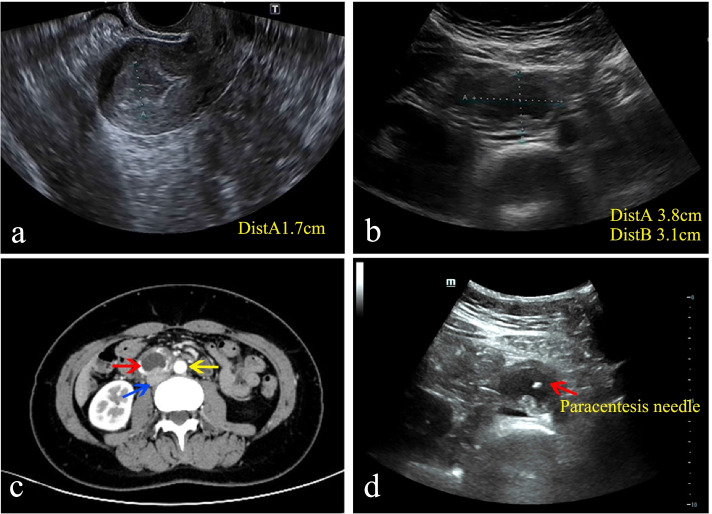
The imaging examination before the laparotomy. **a** Transvaginal ultrasonography (TVS) revealed a thicken endometrium without intrauterine gestational sac. **b** Transabdominal ultrasonography (TAS) revealed a retroperitoneal pregnancy mass. **c** Abdominal computer tomography (CT) showed the retroperitoneal gestational sac (red arrow) was tightly adherent to the inferior vena cava (blue arrow) and abdominal aorta (yellow arrow). **d** Ultrasound-guided paracentesis and local potassium chloride (KCl) injection into the embryo bud

A 29-year-old pregnant woman, gravida 1, para 0, one previous artificial abortion, with regular menstrual cycle, was admitted via the emergency department on December 27 2021 with a history of 50-day amenorrhea and 7-day moderate to intermittent upper abdominal pain. She had no injury history or history of previous pelvic inflammatory diseases or gynecological surgery. Her vital signs were within normal range. General physical examination revealed nothing remarkable. Gynecological examination found no vaginal spotting, and the uterine cervix was smooth without tenderness upon palpation and movement; the uterine body was soft and enlarged equivalent to the size of 50-day-gestation; the right adnexa was slightly thickened without tenderness; and the left adnexa was unremarkable. The serum beta-human chorionic gonadotropin (β-hCG) value was 65,004 m-international units per milliliter (mIU/mL) on admission. Color transvaginal ultrasonography (TVS) of the pelvis demonstrated no intrauterine gestational sac but thicken endometrium of 1.7 centimeter (cm) (Fig. 1a), a right adnexal well-bounded, medially echoic mass approximately 2.3 × 2.0 × 2.0 cm in size with signs of blood supply; no fluid collection in the pouch of Douglas. Because the results of TVS were not parallel with the clinical characteristics and serum β-hCG level, a full transabdominal ultrasonography (TAS) was applied to extend the scan scope. TAS scan revealed a heterogeneous mass approximately 3.8 × 3.1 × 2.3 cm in size, which consisted of a gestational sac with an 4 mm embryo bud with positive cardiac pulsation (Fig. 1b). The pregnancy mass was tightly adjacent to the inferior vena cava and the abdominal aorta. We furtherly completed an abdominal contrast-enhanced computer tomography (CT), which showed the gestational sac with the embryo in the retroperitoneal space and detailed its tight link with the great vessels alongside (Fig. 1c). Highly suspected of rare REP and lack of experience in the diagnosis and treatment of this disease, a multidisciplinary consultation composed of a gynecologist, a vascular surgeon, a radiologist and an interventional physician was scheduled. For fear of vascular injury and unmanageable intraoperative bleeding potentially associated with excising this mass, the patients decided to administer systemic methotrexate (MTX) combined with local potassium chloride solution injection guided by ultrasonography. Daily 20-miligram(mg) intramuscular MTX for 5 consecutive days was initiated on December 28, 2021. And on the same day, ultrasound-guided paracentesis and local potassium chloride (KCl) injection into the embryo bud was operated successfully (Fig. 1d). On December 30 2021, serum β-hCG elevated to 79,382 mIU/ml, but a repeat TAS showed that though the size of REP mass didn’t change, the fetal heart beat was gone. The patient remained stable with close observation in the hospital. Then the medication therapy was continued. However, on December 31 2021, the patient reported worsening abdominal pain and her serum β-hCG level continued to increase (81,447 mIU/ml). Consequently, the patient agreed to undertake an exploratory laparotomy despite stable vital signs and no drop in hemoglobin level (Hb 118 g/L). This was accomplished through a midline incision about 20 cm in length under general anesthesia. While exploring the pelvic cavity, we found a slightly enlarged and soft uterus with bilateral intact fallopian tubes. The left ovary was completely normal while a corpus luteum about 2.0 × 2.0 cm in size was found in the right ovary without active bleeding. No evidence of lesion and pelvic adhesion was found. No fluid collected in the abdominopelvic cavity. Then an abdominal vascular surgeon joined the operation. Further exploration of the upper abdomen revealed a retroperitoneal mass measuring 4.5 × 4.0 × 3.0 cm, inferior the transverse mesentery and directly attached tightly to the surface of inferior vena cava and the left side of abdominal aorta, with a small amount of local retroperitoneal hemorrhage. The retroperitoneal space was entered. After the surrounding connective tissue was carefully dissociated and the communicating vessels between the mass and the inferior vena cava and abdominal aorta were ligated, the pregnancy mass was removed *en bloc*. No blood transfusion was required. The small wound surface on the inferior vena cava was sutured meticulously with absorbable suture to ensure sufficient hemostasis. No retroperitoneal drain was placed. The total blood loss was 50 millilitre (ml) and the operation time was 92 min.

**Fig.2:**
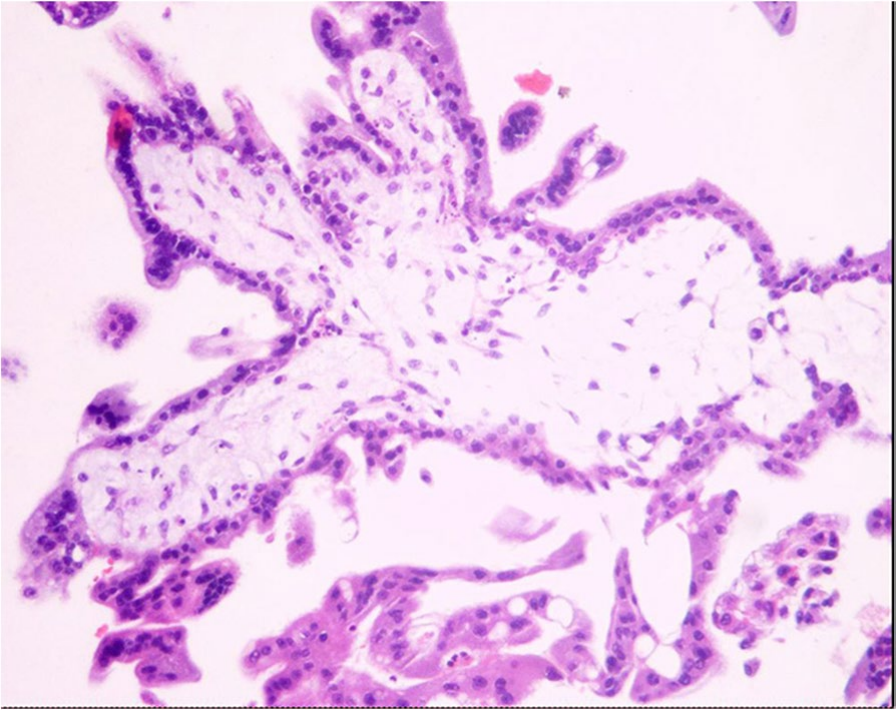
Pathologic examination verified the presence of chorionic villi in the tissue dissected from the retroperitoneal space. Hematoxylin and eosin staining: × 100

An embryo bud was detected macroscopically inside the resected retroperitoneal mass. Pathological examination confirmed the presence of chorionic villi under an inverted microscope (Olympus, Tokyo, Japan) (Fig. 2).

**Fig.3:**
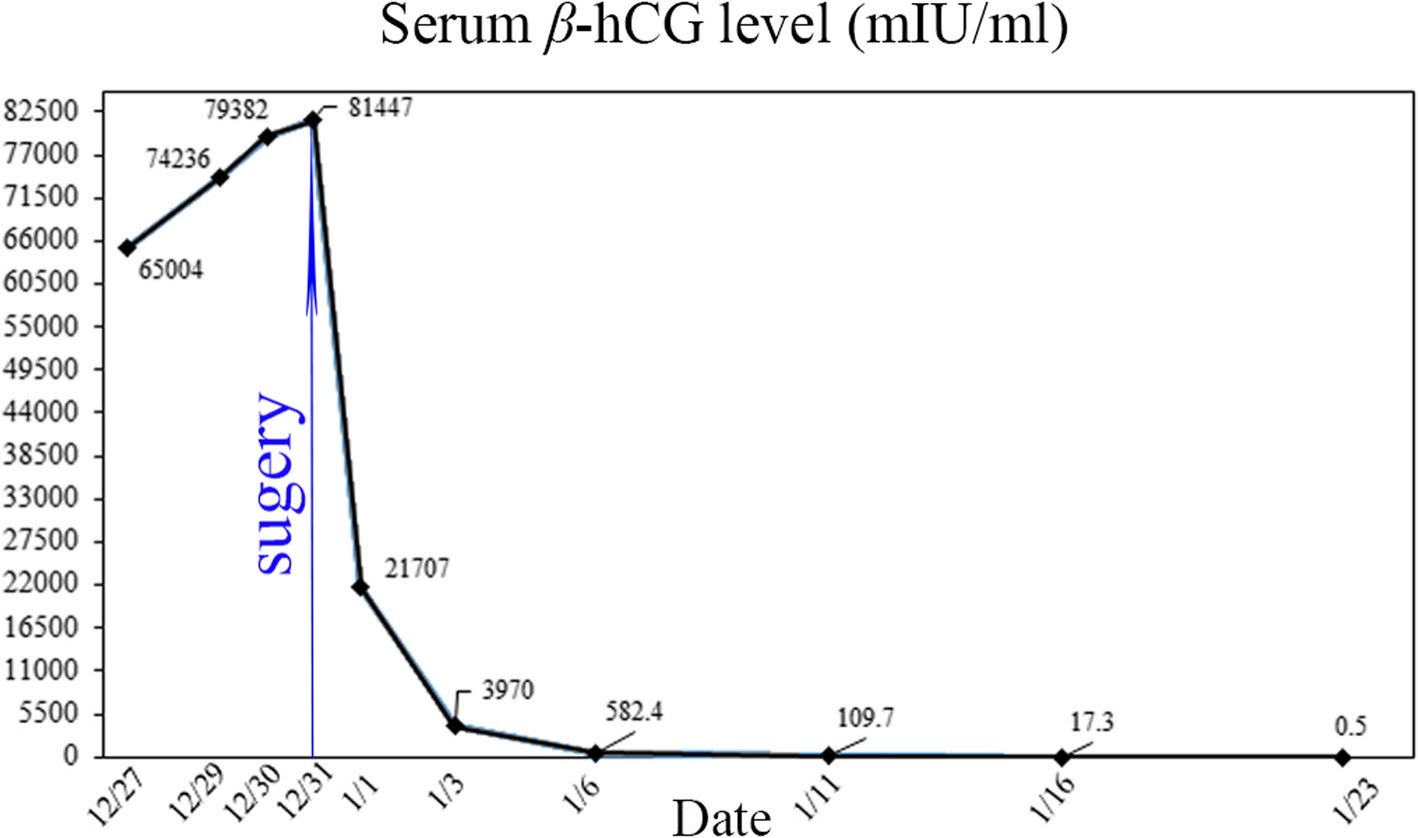
Changes in the patient’s serum beta-human chorionic gonadotropin (β-hCG) levels

Serum β-hCG decreased to 21,707 mIU/mL on the first postoperative day and 582 mIU/mL on the 6^th^ postoperative day. The patient recovered smoothly and was discharged on the 6^th^ postoperative day. Her serum β-hCG were strictly monitored in the outpatient setting and returned to normal range on the 23^th^ postoperative day. Changes in the serum β-hCG levels over time are shown in Fig. 3.

## Discussion and conclusions

Abdominal pregnancy is the rarest type of ectopic pregnancies, possessing eight times higher rates of maternal mortality and morbidity than nonabdominal cases [2]. According to the criteria established by Studdiford in 1942 [6], only a very small fraction of the reported cases could be exclusively diagnosed as primary abdominal pregnancy. Reported common sites of primary abdominal pregnancy are the pouch of Douglas, posterior uterine wall, uterine fundus, anterior abdominal wall, omentum, liver, spleen, and diaphragm [7, 8]. However, abdominal pregnancy in the retroperitoneal space is an exceedingly rare occurrence. Due to its rarity, it is impossible to accurately calculate the incidence of REP. Given its propensity to implant along major vessels, REP poses a high risk of fatal rupture and bleeding. To date, however, there is no well-defined consensus or guideline for clinical management. Bizarre implantation locations, non-specific symptoms and varied clinical presentations can make the diagnosis and treatment of REP challenging, sometimes resulting in misdiagnosis. In order to better guide clinical practice, we conducted a search of PubMed database (English language; 1970–2022; search terms: “retroperitoneal ectopic pregnancy” and “retroperitoneal pregnancy”), and supplemented related cases through literature tracking. A total of 31 literatures including 32 REP cases, plus the case presented here, were collected and thoroughly analyzed, focusing on the clinical characteristics, diagnosis, treatment and prognosis (details listed in Table 1 [4, 5, 9–37]).

**Table 1:** Summary of all reported cases of retroperitoneal ectopic pregnancy in the English literature (*n* = 33)

First author,year	Age,y	Previous normal pregnancy	Previous EP/type	Previous tubal surgery	Pregnancy way/Embryo number	Amenorrhea duration (d)	Primary symptoms	Emergency/shock	hCG before treatment (IU/L)	Auxilliary examinations
Hall, 1973 [4]	21	2	1/tubal pregnancy	Right salpingectomy	SP	35	Left-sided abdominal pain and fainting	Yes/Yes	NA	No
Sotus PC, 1977 [5]	30	3	0	No	SP	73	Vaginal bleeding, persistent left lower-quadrant pain	No	NA	TAS
Ferland, 1991 [9]	32	NA	1/tubal pregnancy	Right salpingectomy	IVF/3	54	Right abdominal pain	Yes/No	19,540	TAS
Dmowski, 2002 [10]	34	0	0	Bilateral salpingectomy	IVF/3	58	Right epigastric and right upper back pain, weakness	Yes/Yes	38,635	TAS/TVS
Reid, 2003 [11]	28	2	3/tubal pregnancies	Bilateral salpingectomy	IVF/3	70	Severe left iliac fossa pain	No	5000	No
Lee, 2005 [12]	21	0	0	No	SP	42	Left flank pain	Yes/No	NA	TAS
Meire,2007 [13]	30	2	0	No	SP	161	Asymptomatic	No	NA	TAS/CT
Iwama, 2007 [14]	31	0	1/tubal pregnancy	Bilateral salpingectomy	IVF/3	49	Slight upper abdominal pain	No	31,778	TAS/MRI
Chang, 2008 [15]	33	2	0	No	SP	44	Progressive lower abdominal pain and 3 episodes of syncope	Yes/Yes	NA	TVS
Lin, 2008 [16]	19	0	0	No	SP	49	Right lower quadrant abdominal pain and vaginal spotting	No	267.31	TAS/TVS/CT
Bae, 2009 [17]	28	1	0	No	SP	54	Vaginal spotting	No	20,328.2	TVS/CT
Persson, 2010 [18]	33	1	1/tubal pregnancy	Right salpingectomy	IVF/2	44	Vaginal bleeding	No	18,032	TVS
Okorie CO, 2010 [19]	28	3	0	No	SP	47	Moderate to intermittent significant lower abdominal pain	Yes/No	NA	TAS
Mart ınez-Varea, 2011 [20]	37	1	0	No	IUI	43	Lower abdominal pain	Yes/No	7787	TVS
Jiang, 2014 [21]	33	2	1/tubal pregnancy	Right salpingectomy	SP	54	mild lower abdominal pain	No	18,920	TVS/TAS/MRI/CT
Liang,2014 [22]	26	0	1/tubal pregnancy	Left salpingectomy	IVF/3	90	left intermittent flank pain	No	1076	TVS/TAS/CT
Protopapas, 2014 [23]	31	1	1/tubal pregnancy	Right salpingectomy	SP	42	Asymptomatic	No	9832	TVS
Ouassour,2017 [24]	35	2	1/tubal pregnancy	Left salpingectomy	SP	49	Asymptomatic	No	6000	TVS/TAS/MRI
Yang, 2017 [25]	32	5	0	No	SP	38	Left lower abdominal pain accompanied with mild nausea, tender breasts, and rectal pressure	No	1880	TVS
Pak,2018 [26]	30	3	0	No	SP	62	Left flank and abdominal pain, vaginal bleeding	Yes/Yes	40,532	No
Yang,2018 [27]	34	1	0	No	SP	52	Bellyache, dizziness, flustered, fatigue, thirsty, and urinary incontinence	Yes/Yes	6803	TVS/CT
Veleminsky, 2018 [28]	38	NA	0	No	SP	49	Asymptomatic	No	33,742	TVS/TAS
Zhang, 2018 [29]	29	NA	0	No	SP	60	Left lower flank pain	No	36,312	TVS/TAS
Huang, 2019 [30]	37	0	0	Bilateral salpingectomy	IVF/2	68	Asymptomatic	No	88,165	TAS/MRI
Huang, 2019 [30]	31	0	1/tubal pregnancy	Right salpingostomy	SP	73	Asymptomatic	No	97,333	TAS/CT
Lu, 2019 [31]	31	1	1/tubal pregnancy	Right salpingostomy	SP	54	Vaginal spotting and lower abdominal pain	No	47,440	TVS/TAS
Wang, 2020 [32]	33	2	3/tubal pregnancies	Bilateral salpingectomy	IVF/2	52	Left back pain, worsening	No	74,678	TVS/TAS/CT
Le,2020 [33]	31	NA	NA	Bilateral salpingectomy	IVF/1	41	Acute epigastric pain	Yes/No	20,625	TVS/TAS/CT
Hou, 2021 [34]	29	1	No	No	SP	49	First asymptomatic, then acute intolerable left abdomen pain	Yes/No	28,746	TVS/TAS/CT
Anh,2021 [35]	34	3	2/tubal pregnancies	Bilateral salpingectomy	IVF/2	51	Vaginal bleeding	No	36,386	TVS/TAS/MRI
Wen, 2021 [36]	28	2	No	No	SP	60	Left lower quadrant abdominal pain	Yes/No	99,286	TAS/MRI
Lorenzo,2021 [37]	33	0	0	No	SP	56	Acute abdominal pain	Yes/No	1053	TVS
This case	29	1	0	No	SP	50	Lower quadrant abdominal pain	Yes/No	65,004	TVS/TAS/CT

First author,year	Implantation site of REP	Size of the lesion (cm)	Embryo/cardiac activity	Initial diagnosis	Initial treatment method	Final treatment method	Rupture of REP	Blood transfusion	Definitive diagnosis
Hall, 1973 [4]	above the bifurcation of the aorta	NA	No	-	Laparotomy/RRP	No	Yes	Yes	Surgical findings and pathology
Sotus PC, 1977 [5]	left side of the aorta and the superolateral side of the left iliac artery	8 × 10	Yes/No	Adnexal EP	Suction, D&C and laparoscopy, unremarkable	Laparotomy/partial RRP	No	No	Surgical findings and pathology
Ferland, 1991 [9]	Upper abdominal retroperitoneal space	NA	No	-	Laparotomy/RRP	No	Yes	No	Pathology
Dmowski, 2002 [10]	Posterior to the duodenum and adherent to the head of pancreas	2 × 3	No	Failing intrauterine pregnancy after IVF	Laparotomy/RRP	No	Yes	Yes	Surgical findings and pathology
Reid, 2003 [11]	The bifurcation of the common iliac artery	6 × 6	No	Miscarriage	Evacuation of uterus	Laparoscopy, converted to Laparotomy/partial RRP	No	No	Pathology
Lee, 2005 [12]	Left paraaortic region below the left kidney	5	Yes/Yes	REP	Laparotomy/partial RRP	No	No	No	Surgical findings and pathology
Meire,2007 [13]	Retroperitoneal paravesical space on the right pelvic cavity	NA	Yes/No	Intrauterine midterm ancephalus	Vaginal induced abortion	Laparotomy/RRP	No	No	Surgical findings
Iwama, 2007 [14]	Adjacent to the aorta and pressed on the inferior vena cava	4.0 × 2.5	Yes/No	Adnexal EP	Evacuation of the uterus, diagnostic laparoscopy and then two-round i.m.MTX(50 mg/m2/per time)	Laparotomy/RRP	No	No	Surgical findings
Chang, 2008 [15]	Retroperitoneal space of the left paracolic sulcus	NA	No	Adnexal EP rupture	Laparoscopy/RRP	No	No	No	Surgical findings and pathology
Lin, 2008 [16]	At the right obturator foramen area	4.0 × 4.0	No	Adnexal EP	Diagnostic laparoscopy and D&C	Laparotomy/RRP	No	No	Pathology
Bae, 2009 [17]	Implanted on inferior vena cava	3.4 × 2.6	Yes/Yes	Cornual pregnancy	Laparoscopic wedge resection of the left uterine cornua and D&C	Laparoscopy/RRP	No	No	Surgical findings and pathology
Persson, 2010 [18]	In the obturator fossa	NA	Yes/Yes	Adnexal EP	First laparoscopic left salpingectomy	Second diagnostic laparoscopy/Robot- assisted laparoscopy/RRP	No	No	Surgical findings and pathology
Okorie CO, 2010 [19]	Overlying the inferior vena cava and the aorta near the second and third parts of the duodenum	3.8 × 4.1 cm then increased to 8.2 × 6.6 cm	No	Adnexal EP rupture	Exploratory laparotomy, then intramuscular MTX(100 mg), HCG declinced but abdominal pain aggravated	Emergent laparotomy/RRP	No	No	Pathology
Mart ınez-Varea, 2011 [20]	Next to the left uterosacral ligament	3.0 × 2.0	Yes/No	adnexal EP	Laparoscopy/RRP	Postoperative i.m. MTX50mg/m2	No	No	Surgical findings and pathology
Jiang, 2014 [21]	Inferior to the duodenum and attached to the surface of the inferior vena cava, as well as the abdominal aorta	6.0 × 6.0	Yes/No	Choriocarcinoma	D&C/intramuscular MTX (daily 20 mg for 5 consecutive days), HCG declined slowly	Laparotomy/RRP	No	No	Pathology
Liang,2014 [22]	Next to the abdominal aorta, ovary vessels and the left renal vein	6.5 × 5.4	Yes/No	adnexal EP	Laparoscopic right salpingectomy, HCG elevated	Laparotomy/RRP	No	Yes	Surgical findings and pathology
Protopapas, 2014 [23]	Retroperitoneal broad ligament	3 × 2.5	Yes/Yes	Cornual pregnancy	Diagnostic laparoscopy	Second laparoscopy combined with hysterscopy guided with transvaginal ultrasound probe	No	No	Surgical findings and pathology
Ouassour,2017 [24]	Attached to the left side of abdominal aorta	6.0	Yes/No	adnexal EP	Exploratory laparotomy	Second laparotmy/RRP	No	No	Surgical findings and pathology
Yang, 2017 [25]	Lateral to the left sacrocervical ligament	2.1 × 2.0	No	adnexal EP	Laparoscopy/RRP	No	No	No	Surgical findings and pathology
Pak,2018 [26]	Retroperitoneal space on the left pelvic cavity	NA	No	休克直接开腹	First emergent laparotomy/ICU	Second laparotomy/evacuated hematoma	Yes	Yes	Pathology
Yang,2018 [27]	In the right lateral abdominal peritoneum region	12.0 × 8.0	No	REP	Laparoscopy/RRP	No	Yes	No	Surgical findings and pathology
Veleminsky, 2018 [28]	Above the vena cava inferior	2.7	Yes/No	Miscarriage/anembryonic pregnancy	Evacuation	Diagnostic laparoscopy/Laparotomy/RRP	No	No	Surgical findings and pathology
Zhang, 2018 [29]	On the left side of the abdominal aorta and encased the left renal vessels	4.1 × 2.9 cm,increasing to 11.0 cm	Yes/No	REP	MTX and selective arterial embolization therapy, HCG elevated	Laparotomy/RRP	No	No	Surgical findings
Huang, 2019 [30]	Under the left renal hilum, in front of the psoas muscle, and to the left of the abdominal aorta	4.2 × 4.2	Yes/Yes	REP	100 mg MTX injecting into the gestational sac under CT guidance	No	No	No	Radiological findings and gestational sac fluid pathology
Huang, 2019 [30]	Adjacent to the left renal hilum, the abdominal aorta, and the IVC, anterior and to the left of the L3 vertebra	4.6 × 3.4	Yes/No	REP	75 mg MTX injecting into the gestational sac under CT guidance	No	No	No	Radiological findings and gestational sac fluid pathology
Lu, 2019 [31]	Adjacent abdominal aorta and inferior vena cava	3.0 × 2.3	Yes/Yes	REP	Laparoscopy/partial RRP	No	No	No	Surgical findings and pathology
Wang, 2020 [32]	Implanted in the left psoas major muscle at the position of the left renal hilum	4.9 × 3.9	Yes/No	Embryo arrest	First D&C for intrauterine embryo arrest	Laparotomy/RRP with 10 mg MTX injected locally	No	No	Surgical findings and pathology
Le,2020 [33]	Attached to the left side of the abdominal aorta	NA	No	REP	Laparotomy/RRP	No	No	No	Surgical findings
Hou, 2021 [34]	Between the abdomi-nal aorta and left common iliac artery	2.7 × 2.5, then6 × 6 cm	Yes/No	adnexal EP	Diagnostic laparoscopy	Emergent laparotomy/RRP	Yes	Yes	Surgical findings and pathology
Anh,2021 [35]	In close proximity to the right common iliac artery	2.5 × 2.0	Yes/No	Intraabdominal EP	Laparoscopic removal of a small abdominal mass/evacuation of uterus	Second laparotomy/RRP	No	No	Surgical findings and pathology
Wen, 2021 [36]	Below the left renal vessels and the abdominal aorta	5.0 × 4.0	Yes/Yes	Intrauterine pregnancy	D&C for induce abortion, HCG increased	Laparoscopy and 50 mg MTX injected locally/RRP	No	No	Surgical findings and pathology
Lorenzo,2021 [37]	At the left posterior parametrium	3	Yes/Yes	adnexal EP	Diagnostic laparoscopy/MTX (50 mg/m2 body surface area), HCG elevated	Second laparoscopy /RRP	No	No	Surgical findings and pathology
This case	Attached tightly to the surface of inferior vena cava and the left side of abdominal aorta	4.5 × 4.0	Yes/Yes	REP	Intramuscular MTX (daily 20 mg for 2 consecutive days), and US-guided local injection of KCl, HCG increased	Laparotomy	No	No	Surgical findings and pathology

*Abbreviations*: *NA* not applicable, *SP* spontaneous pregnancy, *IVF* in vitro fertilization, *hCG* human chorionic gonadotropin, *TVS* transvaginal ultrasonography, *TAS* transabdominal ultrasonography, *CT* computed tomography, *MRI* magnetic resonance imaging, *EP* ectopic pregnancy, *REP* retroperitoneal ectopic pregnancy, *RRP* resection of retroperitoneal ectopic pregnancy, *MTX* methotrexate, *D&C* Dilation & Curettage

### Pathogenesis

The pathogenesis of primary REP is complex and still unelucidated, but three mechanistic hypotheses have been proposed. It is not surprising that the prevalence of ectopic pregnancy is higher following assisted reproductive technique (ART) procedures than in the general population [38]. Tubal pathology, previous tubal surgery, and previous ectopic pregnancy are the major indications for ART and both have been considered as a major risk factor for the ectopic pregnancy [38]. In this proposed mechanism, embryos are placed in the retroperitoneal space due to iatrogenic uterine perforation, or even less likely, through a fistulous tract formed following salpingectomy. Reviewing all the 33 REP cases, 39.4% (13/33) of the patients had a history of tubal pregnancy, of which 10 cases had 1 time, 1 case had 2 times and another 2 cases had 3 times. 48.5% (16/33) of the patients had a history of tubal surgery, of which 7 cases underwent bilateral salpingectomy and 9 underwent unilateral salpingectomy. 30.3% (10/33) of patients were IVF-ET, and 1 case had undergone intrauterine intro-uterine semination (IUI). However, this mechanism was not likely to explain every case with ART operation because the ET procedure was strictly conducted under sonographic guidance. The iatrogenic placement of the embryos in the retroperitoneal space of the mid or upper abdomen can definitely be excluded considering the length of the transfer catheter and the volume of the ET medium [9–11, 14]. Wang et al. [32] speculated that the fallopian tube stumps after resection could be spontaneously reperfused or formed a fistula, creating a possible communication between the uterine and the retroperitoneal cavity. However, in the case reported by Anh et al. [35], both fallopian tube stumps were visible and intact, and detached from the broad ligaments, excluding this explanation. It is also worth mentioning that 16 cases (48.5%) conceived naturally without tubal pathology or resection.

Ferland et al. [9] proposed a second yet not very convincing hypothesis that the embryo implants on the posterior peritoneal surface and reaches a retroperitoneal space by subsequent trophoblastic invasion through the peritoneum. However, there is no direct evidence to confirm this hypothesis.

The third hypothesis suggests that the fertilized ovum may reach the retroperitoneal space via lymphatic system, similar to the metastasis of gynecological cancer, as lymphatic tissue has been found with ectopic masses during postoperative pathological examination [4, 18, 22, 25, 28]. Lymphatic spread may also explain the frequent localization of REPs at the pelvic sidewalls or along the great vessels, corresponding to the known lymphatic drainage from the uterus. This possibility appeared to be the most plausible mechanism in our case for two reasons. First, there was no history of pelvic surgery or tubal pathology before this spontaneous pregnancy, and no abnormal channels were found between the uterus or fallopian tubes and the retroperitoneal cavity. Second, the gestational sac implanted on the inferior vena cava with intact peritoneum overlying it. In addition, the high proportion of cases associated with IVF may be explained by a deposit of a fertilized ovum deep in the endometrium facilitating a subsequent migration into lymph vessels. However, this intralymphatic migration hypothesis is not absolutely persuasive because only a few cases of REP have been reported to be surrounded by lymphatic tissue during pathological examination. The exact pathogenesis of REP is still worthy of further research.

### Clinical characteristics

The age of 33 REP patients was 19–38 years old, with an average of 30.6y. Amenorrhea, abdominal pain and vaginal bleeding are the most common symptoms of REP. Compared with the lumen of the fallopian tube, the space of the retroperitoneal cavity is much larger and more complex, and so the ectopic gestational sac can grow bigger. The duration of amenorrhea was 35–161 days with an average of 56.8d. Due to the good embryonic development, the blood β-hCG before treatment was 267.3–99,286 IU/L with an average of as high as 31,673.4 IU/L. At the same time, over half of the patients (22/33, 66.7%) demonstrated embryo and/or fetal heartbeat on preoperative ultrasound. The size of ectopic pregnancy mass without rupture can even reach 10 cm. Meire et al. [13] reported a case of a retroperitoneal anencephalic fetus terminated at 23 weeks’ gestation. Among the 33 cases, only abdominal pain accounts for 57.6% (19/33), and only vaginal bleeding accounts for 9.1% (3/33). 12.1% (4/33) of them presented both abdominal pain and vaginal bleeding, and another 18.2% (6/33) were asymptomatic. The degree of pain is usually related to whether the pregnancy mass ruptures. And significantly, the region of pain does not fully reflect the implantation site of pregnancy. Only one case, reported by Wang et al. [32], complained of pain in the left lumbar back which might be caused by ectopic gestational sac growth resulting in stimulation of the nerve of the left psoas major muscle.

Theoretically, embryo implantation site should be randomly distributed in the retroperitoneal space. However, in fact, most of the reported REPs located along the great vessels. Ouyang et al. [3] suggested that, according to the implantation site, REP can be simply divided into two types: pelvic REP and abdominal REP. The former refers to the REP in the pelvic segment below the common iliac vessels, accounting for 27.3% (9/33); the latter refers to the REP around the abdominal aorta, the inferior vena cava, and the common iliac artery, accounting for 72.7% (24/33). Given its intimacy with great vessels, REPs pose a significant risk of life-threatening hemorrhage. Among them, 15.2% (5/33) had hemorrhagic shock at the time of presentation, and 15.2% (5/33) had blood transfusion during the operation.

### Diagnosis and differential diagnosis

Due to the nonspecific clinical manifestations and complex pregnancy sites, the diagnosis of REP can be easily overlooked. In general, clinicians tend to focus the diagnosis on tubal pregnancy, without considering the possibility of REP. TVS examination was firstly undertaken in 63.3% (21/33) of the patients, and except 3 cases of rare heterotopic pregnancy after IVF-ET, the others showed thicken endometrium but no sign of intrauterine pregnancy. For those pelvic REPs, such as obturator fossa pregnancy, or uterosacral ligament pregnancy, TVS can easily misdiagnose it as an adnexal ectopic pregnancy. And those REPs in the mid or upper abdomen may be out of reach for TVS, which potentially increases the risk of misdiagnosis. Fortunately, the development of full abdominal ultrasonography, CT scan and magnetic resonance imaging (MRI) provide a strong support for early diagnosis of rare REP [29, 30, 35]. TAS is the most commonly used examination method (66.7%, 22/33), followed by CT (33.3%, 11/33) and MRI (18.2%, 6/33). Ultrasonography is superior to CT and MRI in determining the presence of yolk sac, embryo or fetal heartbeat, whereas the value of CT and MRI lies more in locating the pregnancy site and delineating the relationship between the gestational sac and the surrounding tissues. However, in some emergent situations, the patients (15.2%, 5/33) needed undertaking laparotomy or laparoscopy directly for life saving, and the diagnosis was made through surgical findings or postoperative pathology. Only 24.2% (8/33) were diagnosed with REP at the initial visit. 12 cases were misdiagnosed as an adnexal ectopic pregnancy and underwent laparoscopy, laparotomy or MTX treatment; 5 cases were misdiagnosed as simple failing intrauterine pregnancy and received medical abortion or curettage; 2 cases were misdiagnosed as cornual pregnancy and underwent laparoscopy; one case was misdiagnosed as intraabdominal pregnancy and underwent laparoscopic abdominal mass resection; and one was misdiagnosed as choriocarcinoma and treated by MTX chemotherapy. Therefore, misdiagnosis rate is quite high among REP cases. Several remarkable points need keeping in mind in the process of diagnosis. Firstly, we should closely monitor β-hCG levels and provide ultrasound examination timely. If there is a high β-hCG levels but no intrauterine pregnancy or no evidence of ordinary ectopic pregnancy, the possibility of REP should be considered and immediately investigated further with additional diagnostic procedures, especially for those with history of tubal surgery and IVF. Secondly, when there is a highly suspected of rare ectopic pregnancy, combined auxiliary examinations should be applied to exactly locate pregnancy site. Besides ultrasound, CT or MRI examination would be instrumental for diagnosis. Thirdly, when laparoscopy or laparotomy is taken in case of highly suspected ectopic pregnancy, but no obvious pregnancy mass is found, unusual locations such as the retroperitoneum should be carefully examined. If possible, intraoperative real-time ultrasound guidance may assist in finding the pregnancy site. Last but least, when the patient is hemodynamically unstable and imaging is unavailable, laparotomy only revealed retroperitoneal hematoma but no evidence of hemorrhagic spot, evacuation of retroperitoneal hematoma for histopathology may be helpful for diagnosis.

### Treatment

Due to the high preoperative misdiagnosis rate, 63.4% (21/33) of REP patients have undergone two or more treatments (medication or surgery treatment), of which 6 cases experienced three treatments. Considering the invasive and vascularized nature of the villi tissue and its intimacy with surrounding organs and vasculature, the opinion of a multidisciplinary team is very important and necessary for selecting a suitable treatment program. Surgery is the mainstay in REP management, including laparoscopy and laparotomy. For women with stable haemodynamics, laparoscopic surgery is generally preferred over laparotomic surgery with advantage of shorter operative time and reduced blood loss. However, because REPs are often located alongside retroperitoneal great vessels, laparoscopic resection would be a great challenge. Otherwise, the choice of surgical approach is also related to the experience of the surgeon. Ferland et al. [9] had an attempt of robot-assisted laparoscopic removal of the REP mass implanted deeply in the right obturator fossa and obtained a good prognosis. Before attempting laparoscopic management, radiological examinations such as MRI, color Doppler ultrasonography may be necessary to elucidate the vascular supply of the pregnancy mass and exclude the infiltration of large retroperitoneal vascular, especially in more advanced gestations [18, 36]. Any gynecologist attempting such a procedure should be well-trained, have a thorough knowledge of the retroperitoneal anatomy, and be ready to convert to laparotomy in case of intraoperative complications or uncontrollable bleeding. Close cooperation with an abdominal surgeon and/or an interventional radiologist may prove invaluable to safely carry out these procedures. During the operation, complete resection of REP lesion is the first choice but not always the best, especially when the trophoblastic tissue invades surrounding organs or tissues. Singh Y et al. [39] suggested that the placenta should be preserved locally to avoid bleeding and organ damage caused by stripping, but the disadvantage was that the risk of postoperative infection, secondary bleeding and even trophoblastic disease increased.

Medical management might be a choice for a proportion of patients. Among the 6 cases of systemic treatment with MTX, 3 cases (including our case) chose such medical treatment after diagnosis of REP for fear of vascular injury and massive intraoperative hemorrhage [19, 37], whereas 2 cases were given intramuscular MTX due to misdiagnosis of adnexal ectopic pregnancy and choriocarcinoma, respectively [14, 21], and the other one was given after surgical resection of REP lesion [20]. In our case, ultrasound-guided local injection of potassium chloride solution into gestation sac was combined with systemic MTX in order to reduce embryonic activity. Zhang et al. [29] reported one patient treated with MTX and selective arterial embolization therapy. Unfortunately, all of the 6 cases were finally treated with retroperitoneal pregnancy resection due to treatment failure. Several factors may be responsible for the failure of systemic methotrexate treatment for REP, such as higher blood β-hCG levels, more advanced gestations, and presence of ectopic viable embryo. Remarkably, Huang et al. [30] reported 2 cases of REP who were successfully by CT-guided paracentesis and local MTX injection in the gestational sac. Although surgery is avoided, this method was time consuming for normalization of hCG levels.

MTX can also be used in combination with surgery. Ansong et al. [40] suggested that compared with operation alone, operation combined with MTX (i.m. 50 mg/m2) for abdominal pregnancy could significantly reduce bleeding and shorten the hospitalization time. Therefore, two cases underwent local MTX injection in gestational sac implantation site during the operation, for purpose of killing trophoblast cells, decreasing β-hCG, and reducing relevant complications [32, 36].

There were several limitations existing in our study. Because of the rarity of REP, the number of cases was small. Though reviewed all the included cases in detail, we still can’t figure out a definitive consensus or guideline for the management of REP. Through the case reported here, we emphasize the cooperation of a multidisciplinary team in clinical practice, and a treatment consensus is best devised via input from gynecologists, vascular surgeons, radiologists, interventional physicians, pathologists, and the patient. Besides, only English literature published in PubMed database was included in our study. Many cases reported in other languages or databases must have been missed.

In conclusion, REP is exceedingly rare and its pathogenesis is still unelucidated currently. Due to the non-specific clinical manifestations and complex pregnancy site, REP requires a high index of suspicion to reach a timely diagnosis and management. Abdominal ultrasound, CT and MRI are extremely important in the diagnosis and localization of REP. Although successful conservative treatment has been reported, surgery is still the mainstay in REP management. Given the propensity of REPs to implant alongside great vessels, a multidisciplinary approach and adequate preparation are essential to make a suitable surgical plan to alleviate life-threatening complications.

## Data Availability

All data analyzed during this study are available from the corresponding author on reasonable request.

## Abbreviations

hCG: Human chorionic gonadotropin
REP: Retroperitoneal ectopic pregnancy
TVS: Transvaginal ultrasonography
TAS: Transabdominal ultrasonography
CT: Computer tomography
MRI: Magnetic resonance imaging
IVF-ET: In vitro fertilization – embryo transfer
MTX: Methotrexate
